# Consciousness Under the Spotlight: The Problem of Measuring Subjective Experience

**DOI:** 10.1002/wcs.1697

**Published:** 2024-10-24

**Authors:** Mikel Jimenez, Antonio Prieto, José Antonio Hinojosa, Pedro R. Montoro

**Affiliations:** ^1^ Department of Psychology University of Durham Durham UK; ^2^ Departamento de Psicología Básica I UNED Madrid Spain; ^3^ Instituto Pluridisciplinar, Universidad Complutense de Madrid Madrid Spain; ^4^ Departamento de Psicología Experimental, Procesos Psicológicos y Logopedia Universidad Complutense de Madrid Madrid Spain; ^5^ Centro de Investigación Nebrija en Cognición (CINC), Universidad de Nebrija Madrid Spain

**Keywords:** awareness, awareness measure, consciousness, subjective/objective awareness threshold, unconscious perception

## Abstract

The study of consciousness is considered by many one of the most difficult contemporary scientific endeavors and confronts several methodological and theoretical challenges. A central issue that makes the study of consciousness so challenging is that, while the rest of science is concerned with problems that can be verified from a “third person” view (i.e., objectively), the study of consciousness deals with the phenomenon of subjective experience, only accessible from a “first person” view. In the present article, we review early (starting during the late 19th century) and later efforts on measuring consciousness and its absence, focusing on the two main approaches used by researchers within the field: objective (i.e., performance based) and subjective (i.e., report based) measures of awareness. In addition, we compare the advantages and disadvantages of both types of awareness measures, evaluate them according to different methodological considerations, and discuss, among other issues, the possibility of comparing them by transforming them to a common sensitivity measure (*d*′). Finally, we explore several new approaches—such as Bayesian models to support the absence of awareness or new machine‐learning based decoding models—as well as future challenges—such as measuring the *qualia*, the qualitative contents of awareness—in consciousness research.

## Graphical abstract

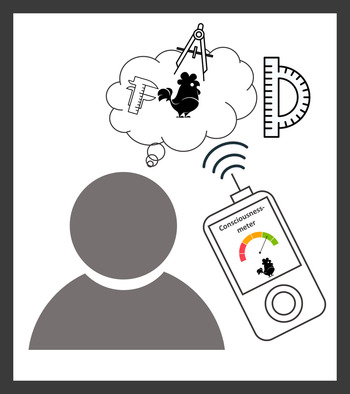

## 1 Introduction

Understanding consciousness is one of the most exciting endeavors of human knowledge. Despite its scientific study has blossomed during the last three decades (Michel et al. 2019), studies on awareness and subjective experience were already being conducted in the late 19th century, at the inception of psychology as a scientific discipline, and continued throughout the 20th century (LeDoux et al. 2020). A fruitful body of research has come since from the study of the relationships between brain activity and consciousness (see Koch et al. 2016; Yaron et al. 2021, for extensive reviews), many efforts being devoted to the search for the “neural correlates of consciousness” (NCCs), the set of neural populations and activities in the brain that are minimally sufficient for bringing about consciousness (Koch 2004).^1^ At the core of all the different methodologies within consciousness research lies a main issue which is the topic of this review: the problem of measuring consciousness. In this article, we will discuss the different approaches and challenges associated with measuring consciousness (or its absence) since the first attempts were laid out almost 150 years ago.

### 1.1 But What Is the Problem? Third Person Versus First Person Data

Any explanation of consciousness faces an inherent problem, termed the “hard problem” by philosopher David Chalmers (Chalmers 1995, 1996), which refers to the impossibility of explaining subjective experience from physical matter. It has been argued that empirical science has a very similar, and maybe more fundamental, “hard problem” (Overgaard 2015a). It relates to the inability to observe subjective states in others: Whereas all behavior and brain processes can be measured from a “third'‐person point of view” by any observer, subjective experiences can only be observed by the individual experiencing them, from a “first‐person point of view.” More generally, while most research fields in science are concerned with problems that can be verified from a “third‐person” view, the study of consciousness deals with the phenomenon of subjective experience, only accessible from a “first‐person” perspective.

**FIGURE 1:**
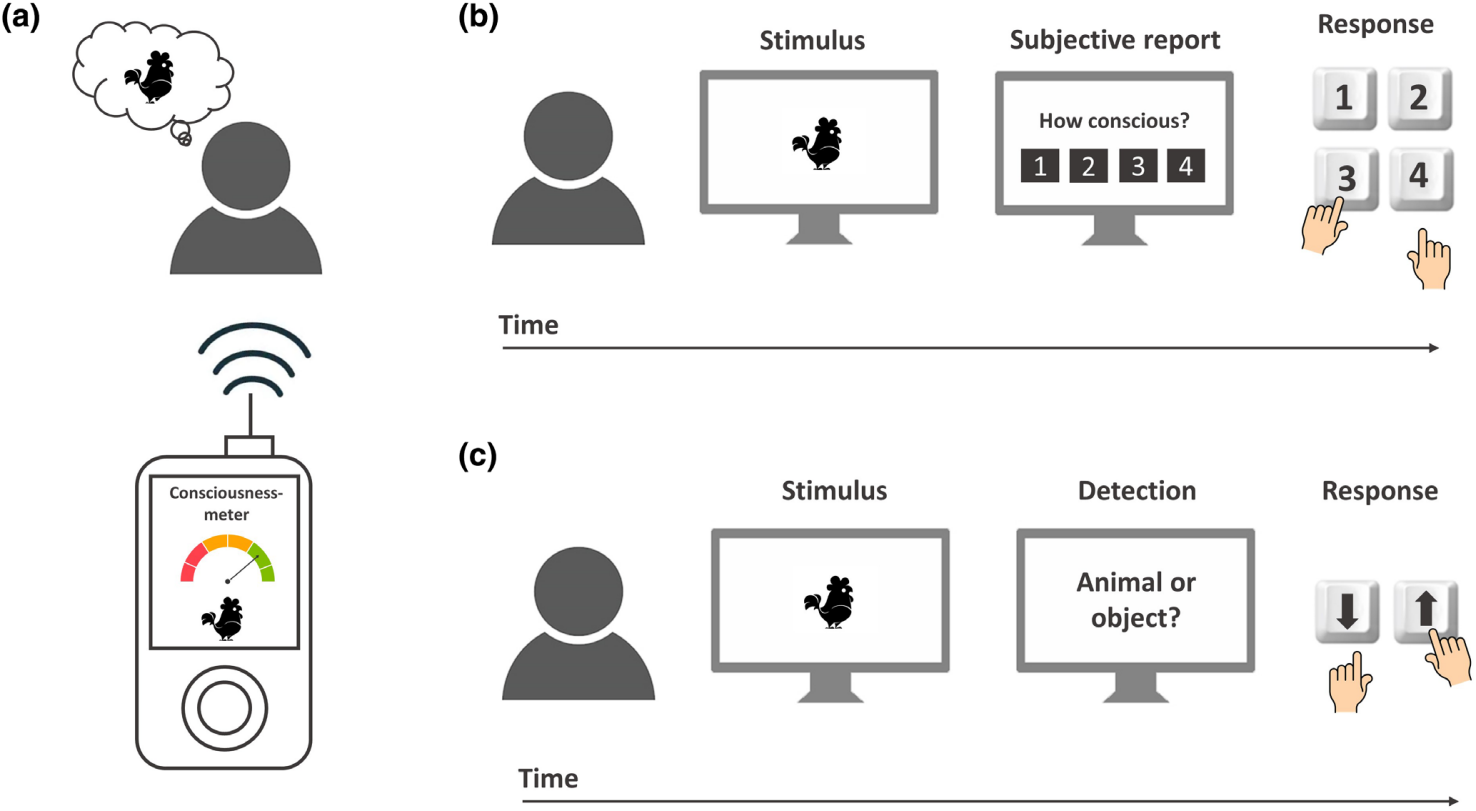
Illustration of (a) a hypothetic “consciousness‐meter,” (b) collection of “subjective” measures of awareness, and (c) collection of “objective” measures of awareness.

Ideally, there would be a scenario where we could acquire the knowledge or technique, such as a “consciousness‐meter,” to transform “first‐person experience” into “third‐person observations” (Overgaard 2015a, see Figure 1a). That would enable the establishment of precise connections among external situations, individuals' subjective experiences, and their observable behavior. However, we do not possess such an instrument and cannot conceive yet a feasible way to develop one (but see Formisano et al., 2008; Haynes 2009; Koch and Mackenzie 2017; Liang et al. 2020). Consequently, linking conscious experience with particular forms of third‐person data, typically different forms of responses (e.g., in controlled laboratory experiments), appears to be the main available alternative.

### 1.2 The “Objective” and “Subjective” Measures of Awareness

Conscious perception has been typically assessed with either objective (performance based) or subjective (experience based) measures of awareness (Persuh 2018; Seth et al. 2008). Observers' reports about their experience (see Figure 1b) are the most direct approach to determining if a person possesses awareness of certain information and is commonly referred to as a subjective measure of awareness. This method, however, is intertwined with potential confounds, such as the inability to report vague experiences or the influence of different decision‐making biases, such as differences in response criterion between participants (some observers adopting a more conservative criterion while others adopting a more relaxed criterion to report a conscious experience) or criterion shifts within participants during the same experimental session (i.e., modifying the criterion to report a conscious experience depending on different factors, such as task demands; see Newell and Shanks 2014, for a comprehensive list of caveats, or Jimenez, Hinojosa, and Montoro 2020, for a discussion on criterion confounds). The problem of response criterion (or the “criterion problem,” see Michel 2023b) poses a crucial challenge to subjective measures of awareness, since it might lead to underestimating or overestimating the observer's true awareness of a particular stimulus. Consequently, subjective methods have been dismissed by many researchers, who instead embraced what is commonly referred to as objective methods for assessing awareness.

Objective methods typically involve instructing individuals to select their response from different alternatives, such as in a two‐alternative forced‐choice task, rather than describing what they perceived or felt (Overgaard 2015b, see Figure 1c). With objective measures, conscious perception is typically estimated from observers' performance in discrimination tasks (Persuh 2018). While objective methods offer the advantage of generating third‐person—objective—data, they are an indirect way (e.g., performance based) to assess observers' awareness. Thus, their validity has been questioned by many authors since it presupposes, unlike subjective methods, that awareness of some information and (behavioral) sensitivity to that same information involve the very same processes, assuming a perfect overlap between performance on a certain well‐defined task and awareness.

Subjective measures of awareness would appear to directly capture the experiential qualities of experience, aligning with the first‐person perspective of *phenomenal consciousness* (Kiefer and Kammer 2024). *Phenomenal consciousness* refers to the subjective experience of sensory and perceptual information, encompassing the qualitative aspects of what it feels like to experience something (e.g., the “redness” of red, the sweetness of sugar, or the experience of pain; Block 1995). On the other hand, objective performance measures may not reflect the content of *phenomenal consciousness* but instead, index the ability to report or respond according to task instructions or goals. This *access consciousness* (Block 1995) involves the availability of information that can be used for guiding our actions and behavior, and is viewed from a third‐person perspective. In practice, there is no consensus that subjective measures more accurately reflect *phenomenal consciousness*. In fact, reporting one's own subjective experience requires *access* to that experience since the sensory experience must be translated into a report (Kiefer and Kammer 2024). Even further, some researchers (Szczepanowski and Pessoa 2007) have argued that objective measures may more directly reflect *phenomenal consciousness*, while subjective measures might actually indicate access consciousness, as they involve meta‐cognitive evaluations of the phenomenal content (see Kiefer and Kammer 2024, for a comprehensive discussion).

Both subjective and objective methods have been employed to measure awareness of a stimulus since experimental studies on consciousness were introduced in the late 19th century. In the following two sections, we will review the methodological challenges and debates associated with the use of these two methods for measuring awareness.

## 2 Measuring the Absence of Subjective Experience

Maybe paradoxically, consciousness was first introduced as an experimental subject through the exploration of unconscious perception, which emerged alongside the development of psychology as a distinct field in the late 19th century.

### 2.1 Early Approaches: Assessing Subliminal Perception Through Introspective Reports

During the late 19th century, several scientists contemplated the idea that mental processes extended beyond conscious awareness.^2^ Influenced by Wilhelm Wundt, introspective reports were regarded as a scientifically valid means of studying mental states during the latter half of the 19th century. This viewpoint posited that all mental states were potentially accessible for conscious reporting. Despite its limitations, this perspective played a crucial role in developing methods to measure various aspects of conscious experience (Kouider and Dehaene 2007).

An early example of the introspective approach comes from a seminal work by Pierce and Jastrow (1884). In their study, participants (the authors themselves) experienced a gentle pressure on a finger followed by a slightly stronger or weaker pressure. Their task was to report which pressure seemed more intense by rating their subjective experience on a scale ranging from 0 to 3, a rating of 0 indicating no preference between the two options. Additionally, they performed a forced‐choice discrimination task to differentiate between the two possibilities. Even when subjects reported null awareness, they could still distinguish between the alternatives with accuracy well above the 50% chance criterion, suggesting the existence of non‐conscious influences on behavior. Sidis (1898) conducted experiments involving cards displaying alphanumeric characters at a distance. Although subjects reported perceiving only a faint, blurred spot, they performed better than chance in both discriminating whether the stimulus was a digit or a letter and guessing its identity in a forced‐choice task. Similar studies using letters and numbers were conducted by Stroh, Shaw, and Washburn (1908) and Coover (1917), whereas line orientations and geometrical figures were introduced by other authors (Baker 1938; Miller 1939; Williams Jr 1938; see Adams 1957, for a review). These discoveries were extended to the auditory domain by Stroh, Shaw, and Washburn and colleagues (1908), suggesting that participants were still able to guess whispered letter names above chance levels despite reporting no subjective auditory perception, whereas studies in psychoacoustics were expanded by Stanley S. Stevens' work in the 1930s (Stevens 1935, 1937). Overall, studies on subliminal perception involved a variety of similar demonstrations contrasting subjective and objective tasks to dissociate awareness and performance during the first half of the twentieth century (Adams 1957).

### 2.2 The Age of Objective Measurement: Methodological Challenges in Demonstrating Absence of Awareness

The methodological approach for measuring participants' experience of a stimulus greatly shifted after Eriksen's (1956) criticism of the scientific literature on subliminal perception. This author strongly opposed the use of introspection as a valid measure of awareness and suggested that subjective measures might merely reflect a participant's response criterion [indicated as *c* in signal detection theory (SDT); see Macmillan & Creelman 1996] instead of truly capturing the observer's subjective experience, and for being especially sensitive to participants' response biases (Eriksen 1956; Jimenez et al. 2019; Kouider and Dehaene 2007).^3^

After Eriksen's review, the need for homogenization and standardization of measures to objectively measure participants' (absence of) awareness became evident. This was mainly operationalized through the classic dissociation paradigm (Reingold and Merikle 1988; see Schmidt and Biafora 2024, for a recent review), which aimed to show differences between a performance measure and an awareness measure on the same stimuli. Arguably, the most popular technique is the masked priming design, where masked primes are presented to participants and they perform a subsequent discrimination task on a probe stimulus (e.g., the performance measure). In a different block, participants' visibility of the primes is assessed by forcing them to discriminate between the primes (e.g., the awareness measure). If participants are at chance‐level in the prime discrimination task, and significant prime effects are found in the masked priming task, it is concluded that the prime's relevant information is processed by the visual system in the absence of awareness.

This approach quickly raised different methodological concerns (Holender, 1986; Kouider and Dehaene 2007; Jimenez et al. 2023; Timmermans and Cleeremans 2015). Perhaps the most relevant relates to the fact that in many studies the aggregated awareness level of the entire sample would not be at a strict chance level (Harris et al. 2011; Jimenez et al. 2018; Muscarella et al. 2013; see Shanks, 2017, for a review), therefore casting doubts to whether the effects found in the performance task were due to some participants consciously perceiving the primes. Additional challenges posed by the dissociation paradigm have been also extensively discussed. The “retrospective assessment” (Shanks and St John 1994) or “immediacy problem” (Newell and Shanks 2014) refer to the difficulty of obtaining simultaneous measurements of performance and awareness. In other words, the awareness measure of not being sensitive to the relevant information does not necessarily imply that information was processed unconsciously during encoding, but that, for instance, it might have been forgotten before being elicited (Timmermans and Cleeremans 2015). The “information or relevance criterion” states that it is crucial to ensure that the information captured by the awareness measure is indeed relevant for task performance. This criterion highlights the need to establish the significance of the revealed information in relation to the task at hand. In addition, both the performance and awareness measures must adhere to the “sensitivity criterion,” meaning they should be equally responsive to the same relevant information. The “problem of post‐hoc data selection” refers to analyzing data only from participants whose awareness scores fall below a specific cut‐off (e.g., chance level in the prime visibility task). This approach introduces a bias because the measurement error is no longer randomly sampled within the subgroup (e.g., *regression to the mean*; Rothkirch et al., 2022; Shanks 2017).^4^ Finally, there is the methodological concern referring to the fact that the absence of statistically significant evidence for awareness is often (mis)interpreted as evidence of absence (Reingold and Merikle 1988; Schmidt and Vorberg 2006, Shanks 2017, see Section 5.1 for a discussion).

The development of different new experimental techniques, such as the regression‐based method proposed by Greenwald, Klinger, and Schuh (1995) or Jacoby's process dissociation procedure (PDP) (1991) tried to overcome the problems associated with the dissociation paradigm,^5^ yet demonstrating cognition in the absence of awareness turned out to be more elusive than expected.

## 3 Measuring the Contents of Subjective Experience

At the turn of the 20th century, subjective measures of awareness regained popularity within consciousness research, a shift partly precipitated by the newly appointed quest for the Neural Correlates of Consciousness (NCC) and by the need to gather observers' subjective experiences more exhaustively.

### 3.1 The Search for the Neural Correlates of Consciousness: The Contrastive Approach

Crick and Koch's (1990) seminal paper laid down the foundations for the scientific exploration of consciousness. It sparked significant interest and research in the field of consciousness studies and established the groundwork for subsequent investigations into the neural basis of conscious awareness. The main research efforts were directed at isolating the “Neural Correlates of Consciousness” (NCCs), the set of neural populations and activities in the brain that are minimally sufficient for bringing about consciousness (Crick and Koch 1998).

Even though Crick and Koch (1990) acknowledged the challenges and limitations in studying subjective experiences and subjective reports, the standard approach behind the NCC consisted of comparing brain activity associated with stimuli that observers report as consciously perceived (e.g., “aware” condition) to brain activity of the same stimuli that observers' report as not being aware of (e.g., “unaware” condition). This “contrastive approach” was proposed by Bernard Baars (1989), who suggested that contrasting conscious and nonconscious processes could help uncover the neural mechanisms and cognitive processes involved in generating conscious experiences. Baars suggested the idea that the most effective way to study consciousness was to maintain similar experimental conditions between conscious and unconscious trials while manipulating consciousness itself as an independent variable. This was accomplished by presenting the stimuli at *threshold*, which renders the stimulus invisible in approximately half of the trials yet still visible in the remaining trials. Threshold stimulus presentation might be accomplished by manipulating its duration, contrast, or the strength with which is masked (Breitmeyer 2015). Thus, instead of creating stimulus‐based conditions in which one measures performance and awareness, the paradigm shifted to creating situations where observers classified the trials as being conscious or unconscious, and researchers then looked at resulting performance and its neural correlates (Dehaene and Changeux 2011; Förster, Koivisto, and Revonsuo 2020; Koivisto and Revonsuo 2010; Rees 2007; Timmermans and Cleeremans 2015; Tononi and Koch 2008).

### 3.2 Is Consciousness Graded? The PAS Scale

Although the experimental logic behind comparing aware (e.g., “seen”) and unaware (e.g., “unseen”) trials would imply that consciousness is binary or “all‐or‐none,” alternative evidence suggests that a percept might gradually emerge into consciousness through increasing levels of subjective experience. Indeed, whereas some studies find a dichotomous transition from unaware to aware visual perception (Asplund et al 2014; Del Cul et al., 2007; Sekar, Findley, Poeppel, Llinás 2013; Sergent & Dehaene 2004), others show that awareness evolves through different intermediate perceptions, and it thus may be characterized as graded (Jimenez et al. 2018, 2019, 2021, Overgaard et al. 2006; Pretorius, Tredoux, and Malcolm‐Smith 2016; Ramsøy & Overgaard 2004; Seth et al. 2008).

To gather observers' intermediate levels of awareness and disentangle whether awareness is graded or dichotomous, different subjective scales have been developed and adopted throughout the last two decades of consciousness research. In several studies (Sergent and Dehaene 2004; Del Cul et al., 2007) a continuous subjective visibility scale with 21 categories was used where only the endpoints were labeled (e.g., “not seen” to “maximal visibility”). Three‐point and 7‐point scales were also used in several studies looking for a graded or dichotomous nature of visual awareness (see Pretorius, Tredoux, and Malcolm‐Smith 2016, for a review). Ramsøy and Overgaard (2004) proposed an alternative 4‐scale point known as PAS (Perceptual Awareness Scale), which gradually became the common subjective measure in subsequent studies. The PAS was developed by presenting very brief simple shape stimuli (colored triangles, circles, and squares, which could appear in three different locations) and asking the observers to assess the clarity of their experience for each aspect of the stimulus (shape, color, and position) by creating their own awareness categories. The authors recommended using a scale ranging from “no experience at all” to “a clear image” as a means of quantifying their subjective experiences. Participants in a pilot experiment as well as five participants in the main experiment all ended up using a four‐point scale with the categories “No experience,” “A brief/weak glimpse,” “An almost clear image/experience,” and “An absolutely clear image/experience.” Even though with some slight variations between studies, these are the four categories that compose the PAS. The PAS is regarded as the most suitable scale to gather intermediate visibility states when compared to other subjective scales.^6^

### 3.3 Alternative Subjective Measures: Metacognitive Scales

One of the most important aspects of consciousness is the capacity to reflect upon our own thoughts, an ability that has been the ground and basis of the higher‐order thought (HOT) theories of consciousness (Carruthers 2017; Lau and Rosenthal 2011; Sherman, Barrett, and Kanai 2015; see Brown, Lau, and LeDoux (2019), for a review). These theories of consciousness argue that first‐order perceptual representations are not sufficient for conscious awareness, instead, consciousness would depend on higher‐order mental representations of oneself being in those particular first‐order mental states (Lau and Rosenthal 2011). This ability to have some representation of a given mental state as the basis of our phenomenology can be operationalized as metacognitive sensitivity (or performance), the ability to retrospectively judge the correctness of our own decisions (Norman and Price 2015; Sherman, Barrett, and Kanai 2015).

To assess metacognitive judgments of participants' own accuracy, experimental designs employing metacognitive scales usually include an objective (or type‐1, using the authors own nomenclature) task (e.g., target detection or discrimination) on which objective performance can be measured, followed by a so‐called type‐2 (subjective) task (Clarke, Birdsall, and Tanner Jr 1959; Pollack 1959) to evaluate the mental state or representation the subject has of the stimulus (Sherman, Barrett, and Kanai 2015). Two types of judgments have been usually employed when performing a type‐2 (subjective) task to measure metacognitive accuracy, confidence ratings (CR) and post‐decision wagering (PDW).

#### 3.3.1 Confidence Ratings

**FIGURE 2:**
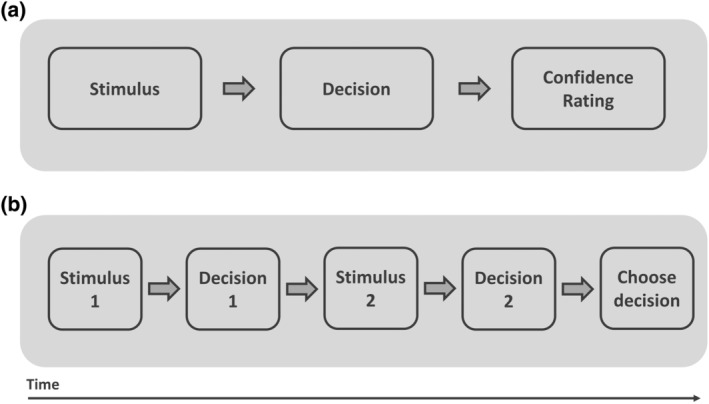
Examples of (a) typical trial sequence in an experiment using confidence ratings (CR), and (b) trial sequence for the 2IFC paradigm. Adapted from Mamassian (2020).

Confidence ratings (CR) are the most widely employed metacognitive judgments in consciousness research and have been extensively used in different experimental contexts like implicit learning, memory, and visual awareness. Their use dates back to the 1980s when Cheesman and Merikle (1984, 1986) introduced them as a way to operationalize the participant's subjective threshold of consciousness. They consist of a retrospective self‐report rating of one's confidence in a decision after that decision has been made (see Figure 2a), and can be provided in different forms such as binary scales (e.g., “less confident” vs. “more confident” or “guess” vs. “sure”), multiple discrete scales (e.g., “(1) not confident at all, (2) slightly confident, (3) quite confident, and (4) very confident”), or continuous scales (e.g., percentage scales from 0 to 100; see Norman and Price 2015, for a review). The main assumption in CR is that they reflect (or at least strongly correlate with) what Ned Block (1995) termed *phenomenal consciousness*, or the subjective experience of being in a particular state. This *phenomenal consciousness* in HOT theories is synonymous with the high‐order mental representations of oneself being in a particular mental state (Lau and Rosenthal 2011). However, CR are not free from controversy. While some authors endorse their use due to their clear understandability, the close relationship to performance on the primary task, the ability to evade the criterion‐content problem, or its comparability across experiments, subjects, and modalities (Dienes and Seth 2010b; Morales and Lau 2021; but see Michel 2023a; and Norman and Price 2015 for a thorough revision), others have criticized them and argued in favor of more direct introspective measure like PAS. In this line, Sandberg et al. (2010) provide empirical evidence to argue that PAS is a more exhaustive and sensitive measure of awareness (see footnote 6, Section 4.3. for further discussion). These authors also claim that directly asking about perceptual experiences offers a better way of collecting information about conscious content (see also Rosenthal 2019). Other authors, however, maintain a somewhat divergent position. For example, Rausch, Müller, and Zehetleitner and colleagues (2015) found that confidence reports are more efficient in predicting the accuracy of the task. According to the authors, the most plausible explanation for this result is that confidence judgments may depend heavily on the conscious access to the relevant feature for the task at hand, while the quality of the visual experience is affected by the conscious access to task‐irrelevant features, which also explains the more restrictive criteria employed in visual experience judgments. In any case, and regardless of the mixed evidence found in the literature, the fact that in CR the observers assess their *conscious* confidence, or the confidence they consciously experience, implies they are not free from the general problems of every subjective report (Rosenthal 2019). Particularly, they are subjected to cases of conscious experiences without confidence or, on the contrary, confidence not based on a subjective experience (Caziot and Mamassian 2021; Koriat 2011).

#### 3.3.2 An Alternative for Measuring Confidence: The Confidence Forced‐Choice Paradigm

The confidence forced‐choice paradigm, also known as the two‐interval forced choice (2IFC) paradigm, has been recently proposed as an alternative method to study confidence (Barthelmé and Mamassian 2010; Mamassian 2016, 2020). In the 2IFC paradigm, the observers are prompted to choose among two perceptual decisions the one they think is more likely to be correct. In a typical experiment (e.g., Peters and Lau 2015), the observers make two perceptual decisions (e.g., reporting the orientation of a masked grating) on two sequentially presented stimuli (see Figure 2b). Then, they decide which of the two perceptual decisions they think is more likely to be correct. The experiment goes on asking participants what they perceive when stimuli are presented, and for every two perceptual decisions, participants are prompted to choose which of the last two perceptual decisions they think are more likely to be correct (de Gardelle and Mamassian 2014; see Mamassian 2020, for a thorough review).

Two‐interval forced choice (2IFC) paradigms are believed to be largely free from biases (Mamassian 2020; Michel 2023b). Since the target is presented randomly in either the first or second interval, any tendency to choose one of the two intervals more frequently will not shift the results in a specific direction. In addition, because the observer's judgment is based on a relative visibility comparison rather than a general visibility judgment, 2IFC paradigms offer relatively clear task instructions. Lastly, considering the stimuli presented in the two intervals differ only in one feature (that's it, the feature affecting target visibility), observers are not required to use multiple criteria or decide how to weight different stimulus features (Michel 2023b). 2IFC paradigms have been criticized based on the so‐called *criterion content fallacy*: mistakenly concluding that subjects are conscious of task‐relevant features simply because they are conscious of *something* (Michel 2023b; Rajananda, Zhu, and Peters 2020). This fallacy does not only apply to 2IFC paradigms but also to studies using retrospective awareness reports (e.g., PAS). Indeed, trials in which subjects report seeing just a “brief glimpse/weak perception” are commonly taken as “conscious trials” by many researchers (e.g., Bergström and Eriksson 2014; King, Pescetelli, and Dehaene 2016; Soto, Mäntylä, and Silvanto 2011; Trübutschek et al. 2019). However, experiencing a “weak perception” of the stimulus is not incompatible with unconsciously representing the task‐relevant features of the stimulus, and it may be even compatible with the complete absence of perception of the task‐relevant features (Michel 2023b).

Follow‐up developments have attempted to resolve the *criterion content fallacy* in a further extension of the 2IFC paradigm (e.g., Amerio et al. 2023; Elosegi, Mei, and Soto 2024; Rajananda, Zhu, and Peters 2020). Within this new version of the 2IFC, a stimulus is presented in both intervals, but only one of the intervals contains the task‐relevant feature. When participants demonstrate above‐chance discrimination in situations where they rate the visibility of the feature as the same in both feature‐present and feature‐absent intervals, the presence of unconscious perception is asserted. Above‐chance discrimination shows that participants processed the task‐relevant feature, while their visibility judgments indicate that, for them, seeing the feature felt indistinguishable from not seeing it (Amerio et al. 2023).

#### 3.3.3 Post‐Decision Wagering

To avoid the limitations of CR, Persaud, McLeod, and Cowey (2007) and Persaud and McLeod (2008) proposed a new test of consciousness in which participants are asked to place wagers on the outcomes of their perceptual decision after that decision has been made, and cash rewards are offered for getting it right. Similar to the CR, the wagers placed by the participants can be binary (e.g., “£1 or £2”; Persaud and McLeod 2008) or multiple categories (e.g., “€5, €10, €15 or €20”; Sandberg et al. 2010). The logic behind Post‐Decision Wagering (PDW) is as follows: if participants perform above chance in the type‐1 objective task but their bets do not distinguish between correct and incorrect decisions (they do not maximize earnings), that indicates a lack of awareness that their decisions are correct, and thus the presence of unconscious processing. The main advantages of using PDW as a measure of consciousness are: (1) PDW exploits people's desire to make money, increasing motivation and reducing the problem of conservative response bias, in which participants tend to withhold the knowledge they held with low confidence; (2) participants find the task more straightforward, intuitive and natural than other subjective reports; and (3) PDW do not alter the conscious states that measure (Persaud et al. 2007; Persaud and McLeod 2008). Despite the authors' claims that it constitutes an objective measure of consciousness, several objections have been raised against its validity and sensitivity as a measure of consciousness from both an empirical and a theoretical point of view. The first one refers to the reluctance to wager from subjects who are loss averse, even when it is advantageous (Dienes and Seth 2010a; Schurger and Sher 2008).^7^ The second objection is related to the authors' claim that PDW is an objective measure of awareness (Persaud, McLeod, and Cowey 2007; Persaud and McLeod 2008). Contrary to that statement, Seth (2008) replicates that PDW is an effective method for assessing metacognitive content, but it is by no means an objective measure of an ontologically subjective phenomenon like consciousness, an argument shared by Rosenthal (2019), who points out that, once all objections against its use have been controlled, PDW constitutes an indirect measure of the contents of consciousness very similar to the ordinary confidence judgments.

## 4 Contrasting Objective and Subjective Measures of Consciousness

As discussed earlier, both the use of objective and subjective measures of awareness comes with their own methodological caveats. In the present section, we will evaluate objective and subjective measures according to different methodological considerations.

### 4.1 Objective and Subjective Thresholds of Awareness

**FIGURE 3:**
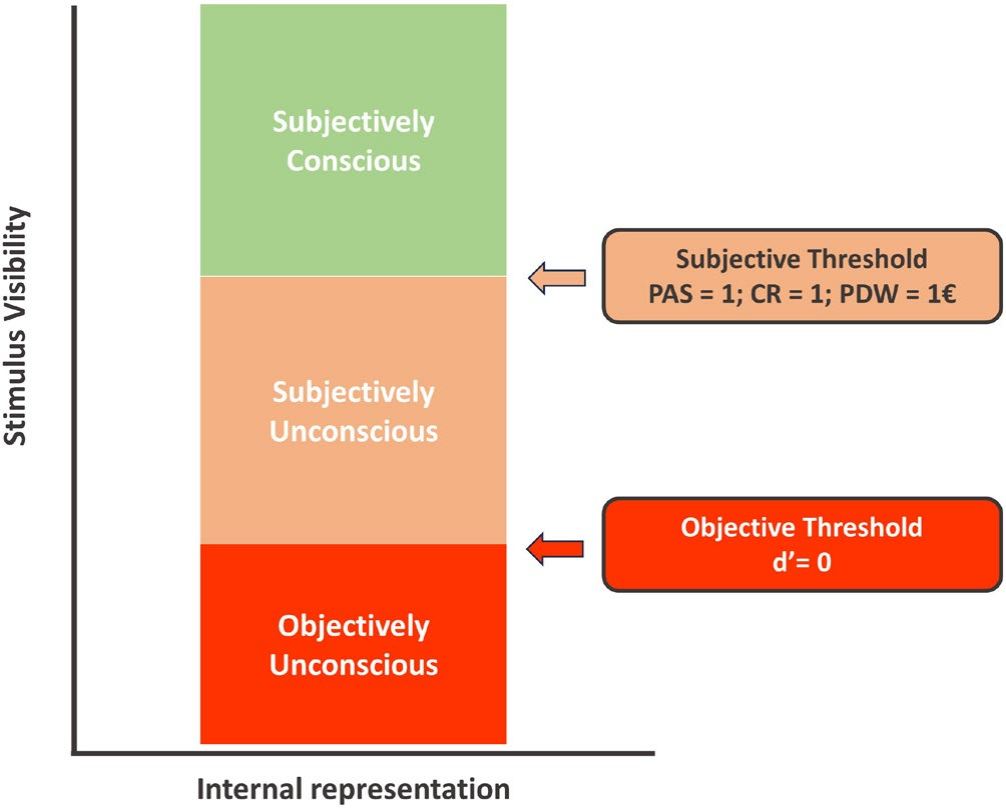
Subjective visual experience as a function of objective and subjective thresholds of awareness. Adapted from Lamme (2020).

*Awareness thresholds* are defined as the minimum stimulus conditions needed to consciously experience a stimulus (Merikle, Smilek, and Eastwood 2001). Interestingly, this *threshold* delineating the boundary between conscious and unconscious states differs between objective and subjective measures of awareness. When using a subjective measure, such as asking participants to report their visual experience, the absence of observer's awareness is assumed for any experience that lays under a *subjective threshold*, in other words, we infer participants unawareness of the stimulus when they report *no perception* of the stimulus (Timmermans and Cleeremans 2015). When using an objective measure of awareness, such as asking participants to discriminate between two alternative stimuli, the absence of stimulus awareness is derived from an *objective threshold*, in this case, the inability to discriminate between the alternative stimuli (e.g., chance performance in the stimulus discrimination task). It is commonly assumed that objective thresholds produce a more conservative estimate of participant's awareness than the subjective threshold, and it has been therefore argued that above‐chance performance in objective awareness measures might not indicate conscious perception but unconscious stimulus influences (Merikle, Smilek, and Eastwood 2001). Interestingly, the distinction between objective and subjective thresholds allows to differentiate several stages in the visual processing of a particular stimulus (see Figure 3): an *objectively unconscious state* (e.g., both objectively and subjectively unconscious, or *fully unconscious*), or those stimuli that are below the threshold for objective visibility; a *subjectively unconscious state*, referring to subjectively unseen stimuli (as reported in PAS, meta *d*′ [see Section 4.4 for a thorough discussion], confidence ratings, etc.) with above threshold performance for objective invisibility (*d*′ > 0); and a *subjectively conscious state*, referring to those stimuli above the objective (*d*′ > 0) and subjective (e.g., PAS > 1, meta *d*′ > 0) thresholds of awareness (Lamme 2020; Figure 3).

Interestingly, recent functional magnetic resonance imaging (fMRI) results (Stein et al., 2021) have associated subjective and objective awareness states with different brain correlates. This new evidence suggests that although both objectively and subjectively invisible stimuli are represented in the visual cortex, the extent of unconscious information processing is influenced by the measurement method (e.g., objective or subjective). For subjectively invisible stimuli, similar to visible stimuli, a clear posterior‐to‐anterior gradient in visual cortex is observed, with stronger category information in ventrotemporal cortex than in the early visual cortex. For objectively invisible stimuli, however, category information remains virtually unchanged from the early visual cortex to object‐ and category‐selective visual areas. Even though further evidence is needed in the brain‐based activations associated with objective and subjectively unconscious mental states (see Section 5.5. in the present article for a further discussion), this approach might prove relevant in the future for the refinement of the current neural theories of consciousness.

Note that the classical outline for awareness thresholds outlined in Figure 3 endorses the view of a linear development of consciousness states, ranging from objectively (or fully) unconscious, over subjectively unconscious (but objectively conscious) up to a subjectively conscious state that also includes an objectively conscious state. This proposed linear progression of consciousness states might be, however, oversimplified and even inconsistent with recent literature. For instance, Kiefer, Frühauf, and Kammer (2023) found a convergence of thresholds for objective and subjective measures of awareness, and a similar pattern has been also observed elsewhere (Jimenez et al. 2023; Schmidt and Biafora 2024). Furthermore, as outlined by Kiefer and Kammer (2024), the relation between subjective and objective measures might be complex and heterogeneous, and in some instances, thresholds of subjective measures can even precede those of objective measures.^8^ As Kiefer and Kammer (2024) discuss, this heterogeneity most likely reflects methodological differences between studies. For example, depending on the specific task, objective measures may be influenced to varying degrees by unconscious response tendencies. This brings up the question of the exclusivity of a measure, which is addressed in the next section.

### 4.2 Are Subjective and Objective Measures Optimally Exhaustive and Exclusive?

As with any rigorous measurement, an ideal measure of consciousness should be both *exhaustive* and *exclusive*. An *exhaustive* measurement has enough categories so that all the observations fall into some category. In other words, *exhaustiveness* refers to the capacity of any measure to capture even the most minimal presence of the measured variable. Therefore, an *exhaustive* measure of consciousness needs to be sensitive to all levels of conscious processing. Along with *exhaustiveness*, an ideal measure of consciousness should also be *exclusive*. *Exclusiveness* means that the categories must be distinct enough that no observations will fall into more than one category. An exclusive measure of awareness should only reflect conscious processing, without contamination by any kind of unconscious knowledge (Timmermans and Cleeremans 2015; Schmidt and Biafora 2024).

*Exhaustiveness* in subjective measures faces a difficult problem, as it must be assumed that every mental state is at least potentially accessible to conscious report. In other words, we must assume that any failure to report knowledge derives from the absence of such knowledge. However, that is far to be true, as failure to report could also reflect a conservative response criterion (Björkman, Juslin, and Winman 1993; Eriksen 1956). Therefore, the inability to inform about knowledge could be reflecting only very low confidence in that knowledge, not the absence of it. This brings us to the main problem with *exhaustiveness* in subjective measures: the absence of evidence of the presence of awareness can never be taken as evidence of its absence (Timmermans and Cleeremans 2015). Objective measures on the other hand would be closer to being exhaustive, as we can assume that the presence of conscious processing will increase performance above the chance level (*d*′ > 0), but under this scenario adequate statistical power is a necessary condition to reduce the probability of type 2 error, an issue already found in previous research (Lin and Murray 2014). In any case, objective measures do not meet the *exclusiveness* criterion, as above‐chance sensitivity may both reflect unconscious perception or residual (weak) stimulus perception, making it impossible to know with absolute certainty what is actually being measured (Merikle and Daneman 1998). The same applies to subjective measures, as the introspective reports could be influenced by unconscious processing, resulting in responses that are influenced by both conscious and unconscious knowledge. Another issue regarding *exclusiveness* and subjective measures is confabulation, or the tendency of participants to justify their choices by appealing to knowledge they do not really have (Johansson et al. 2006), and that may be influenced by unconscious processes.

### 4.3 The Question of Validity: Do All the Scales Measure the Same Underlying Construct?

Even if we were to assume that all the different measures are *exhaustive* and *exclusive* with respect to what they are intended to measure, we would still have to consider whether they are measuring the same phenomena. This is particularly evident in the distinction between objective and subjective measures. While objective measures typically take any behavioral evidence of above‐chance sensitivity (e.g., *d*′ > 0) as evidence of conscious processing, subjective measures go a step further and assume that conscious processing is the sensitivity to a feature that *feels like something* for the observer (Michel 2023a). So, for the issue at hand, the question is: is it possible to have sensitivity in the absence of a *feels like something* experience? Although there is no definitive answer, some studies point to the existence of dissociations between both types of measures (Szczepanowski and Pessoa 2007). The same problem arises when we compare different subjective measures, such as visibility and confidence judgments. Visibility procedures appear, at first glance, to be more straightforward measures, in that they appeal to the clarity of the perceptual subjective experience as a measure of the awareness of that experience (Ramsøy & Overgaard, 2004; Sandberg and Overgaard 2015). In contrast, confidence judgments and other metacognitive measures, rely on providing confidence estimates about the observer's perceptual decisions and, therefore, they measure a second‐order ability to think about your own mental states.^9^ Dissociations between these two measures have been found in several studies (Sandberg et al. 2010; Song, Koizumi, and Lau 2015; Wierzchoń et al. 2014), and there is an ongoing debate between advocates of both measures regarding their validity (see Abid 2019; and Michel 2019, 2023a, 2023b). For example, Sandberg et al. (2010) compared different subjective awareness measures (PAS, CR, and PDW) in a masked visual identification task in which all three scales were presented in the same manner (i.e., a 4‐point scale) and differed only in the scale‐specific instructions employed. Their results indicated that PAS was the most exhaustive scale and showed a stronger performance‐awareness correlation, followed by CR and PDW. The authors interpreted these results in a quite straightforward way: When using a subjective measure of awareness, participants tend to do exactly as instructed. Thus, when asked to report their confidence or to place a wager on their decision, they will not report directly about their experience of the stimulus, even though both are strongly correlated. Similarly, Wierzchoń et al. (2014) compared different subjective awareness scales (PAS, CR, PDW, and *Feeling of warmth* or FOW) in a visual identification task. In line with Sandberg et al. (2010), they found that PAS was the most exhaustive of the four scales compared, but only when awareness reports were given after the identification task. When the awareness ratings were provided before the visual identification task, CR appeared to be the most exhaustive scale. Interestingly, the authors interpreted these results in the light of higher‐order theories of consciousness (HOT, see Section 3.3.). Indeed, the fact that PAS reports are affected by the task order, when they should not if we assume that they are a direct measure of observers' visibility, suggests that PAS ratings benefit from knowledge about the action performed, *de facto* placing it as another metacognitive measure. Hence, measurement divergences between subjective scales might emerge because of their differential sensitivity to different parts of the consciousness spectrum. In summary, these and other recent works (see Michel 2023a, for a thorough comparison between visibility and confidence measures) highlight the need for extensive research into the processes that underlie, and the potential different factors that affect (e.g., the timing of the judgment, the instructions given, or the task at hand), the different measures of awareness.

To outline all the different methodological issues discussed to this point, the strengths and weaknesses of objective and subjective measures of awareness are presented in Table 1.

**TABLE 1:** Methodological strengths and weaknesses of objective and subjective measures of awareness.

Awareness MEASURE	Strengths	Weaknesses
Objective	Produces third‐person, objective, data	Indirect approach to observer's awareness
		Retrospective assessment/immediacy problem
		Information or relevance criterion
		Post hoc data selection
		Affirming the null
		Exclusiveness problem
Subjective	Direct approach to observer's awareness	First‐person, objectively inaccessible data
		Influenced by response criteria (c)
		Criterion content fallacy
		Exhaustiveness problem
		Exclusiveness problem

### 4.4 *When* to Measure Awareness? “Online” Versus “Offline” Approaches

When is the best *time* to obtain a measure of consciousness? In studies investigating unconscious cognitive processes, measures of awareness have been typically collected in a separate block after the main experimental task. This raises the concern of “retrospective assessment” (Shanks & St John 1994) or the “immediacy problem” (Newell and Shanks 2014, see Section 2.2; see Section 3.3 of the present article). Recently, this *offline* procedure has been further criticized (Bengson & Hutchison, 2007; Lähteenmäki et al. 2015) for not being sensitive to intraindividual fluctuations in visual awareness over the many trials (hundreds of them) of an experiment, and alternative *online* measures of prime awareness have been proposed.

*Online* measures involve assessing participants' awareness on a trial‐by‐trial basis, commonly using a subjective measure of awareness (e.g., PAS: Sabary et al., 2020; Kiefer et al. 2023). Following this logic, unconscious and conscious trials can be computed separately during the statistical analysis, and fluctuations of consciousness throughout the experiment can be assessed. However, the inclusion of trial‐wise awareness measures also comes with its downsides, especially when introduced within a masked priming paradigm (Jimenez et al. 2023; Kiefer et al. 2023). Indeed, the implementation of trial‐wise evaluation of awareness *de facto* converts the priming task into a dual‐task paradigm, which may notably affect the reliability of both the indirect measure of priming (probe responses) and the direct assessment of the visibility of the masked stimulus (priming visibility). Among other consequences, dividing participants' attention between prime and probe leads to a slowing of probe latencies (Jimenez et al. 2023; Kiefer et al. 2023), which would interfere with the validity of the response time as a dependent variable. Additionally, it induces a conservative awareness criterion (c) in the use of the *online* subjective scale, most likely due to the high cognitive demands of the dual‐task design and/or participants' being unable to direct their attention to both the prime and probe displays. As a consequence, prime visibility may be biased toward *unaware* reports. On the other side of the coin, accessing prime information seems to be easier, and hence the awareness of the primes higher, when participant's attention is exclusively focused on the prime display as happens when measuring awareness *offline* (e.g., on a separate block). Thus, the visibility of the prime in a classical priming task might be lower than the visibility of the prime inferred from the offline prime visibility task, or in other words, the dissociation paradigm might be *overestimating* actual prime perception (see Jimenez et al. 2023, for a discussion).

The problem of *when* to measure participants' awareness is an ongoing methodological debate. As reviewed in this section, both *online* and *offline* approaches have their own upsides and downsides, and the use of any of those would greatly depend on the type of study and the specific experimental paradigm to be implemented. One avenue to find a solution for this problem comes from the possibility of combining both *online* and *offline* procedures to combine the strengths of both approaches within the same experimental paradigm. Among these lines, Jimenez et al. (2023) recently developed a novel paradigm to explore unconscious processing, in which objective and subjective measures of awareness were introduced at different moments and under different task demands. Using a masked priming design, participants gave their speeded response to a probe stimulus (diamond or square shapes) that was preceded by a masked congruent or incongruent (e.g., a diamond or square shape) prime composed of local elements. This paradigm allowed the authors to evaluate the unconscious processing of the primes under single and dual‐task conditions, employing online/offline and objective/subjective measures of awareness, and under different attentional resources devoted to the primes: no attention (first block), divided attention (second block), and full attention (third block). In a subsequent work, Prieto et al. (2024) refined this paradigm by including both objective and subjective online measures of awareness of the prime stimulus during the second block (dual task), unifying both measures into a single motor response given by the participants in each trial after responding the probe stimulus. This and other alternative approaches combined with novel analysis methods (Section 5) might produce interesting insights in future studies.

### 4.5 Comparing Objective and Subjective Measures by Transforming Them Into a Common Sensitivity Measure (*d*′)

Sensitivity measures (*d*′) based on the signal detection theory (SDT) have been arguably one of the most common tools to assess observers' awareness using objective measures of awareness (Michel 2023a). This is what has been called type‐1 (objective) SDT, usually employed to calculate objective task performance for binary decisions (Green and Swets 1966; Macmillan and Creelman 2004). Type‐1 SDT relies only on objective performance and many researchers were concerned about its capacity to index consciousness exclusively, especially given the methodological issues discussed in Section 4.2 (Holender, 1986; Kouider and Dehaene 2007; Jimenez et al. 2023; Timmermans and Cleeremans 2015).

Thus, some researchers (Kunimoto, Miller, and Pashler 2001; Maniscalco and Lau 2012, 2014) extended the logic of SDT to a version that included second‐order judgments, or type‐2 (subjective) SDT. In this method, participants perform a detection/identification task (type‐1, objective) along with a subjective type‐2 task (a confidence or awareness rating, i.e., subjective), and awareness of their own performance is assessed by how well confidence/awareness judgments track type‐1 decisions (Sandberg and Overgaard 2015). In type‐2 (subjective) tasks, variables are computed in the same way as type‐1 (objective) variables, but instead of signal‐response correspondence, accuracy and awareness/confidence judgments are compared. Particularly, type‐2 hits are defined as correct responses (i.e., a trial in which the stimulus was present, and participants indicated some level of confidence or awareness of the stimulus through a subjective measure), whereas type‐2 false alarms are defined as incorrect responses (i.e., a trial in which the stimulus was absent and nonetheless participants indicated some level of confidence or awareness of perceiving the stimulus). Type‐2 (subjective) d’ is then calculated in the same way as type‐1 (objective) d’. After it was first proposed by Kunimoto, Miller, and Pashler (2001), this measure raised high expectations as it was supposedly free of contamination from response bias. In fact, it has been already employed in several other studies (Jimenez et al. 2023; Kiefer et al. 2023; Lähteenmäki et al. 2015; Szczepanowski and Pessoa 2007). Nonetheless, this absence of bias does not seem to be sustained either empirically or theoretically, as shown by simulations and systematic analyses which indicate that both type‐2 *d*′ and ROC curves are highly dependent on decision and confidence thresholds (Barrett, Dienes, and Seth 2013; Evans and Azzopardi 2007). Also, problems with the general validity of the underlying assumptions have been suggested, especially the Gaussian distribution of the correct and incorrect decisions (Galvin et al. 2003; Maniscalco and Lau 2012). Therefore, special caution must be taken when interpreting the results of type‐2 (subjective) SDT measures (see Barrett, Dienes, and Seth 2013; Evans and Azzopardi 2007 and Sherman, Barrett, and Kanai 2015 for a thorough discussion on signal‐detection and subjective/metacognitive models).

One of the main issues related to the application of the type‐2 (subjective) *d*′ is that it can be influenced by type‐1 (objective) sensitivity (*d*′) and/or response bias (c). This situation may lead to the situation where two optimal metacognitive observers differ in type‐2 (subjective) performance just because they differ in their type‐1 (objective) accuracy, and not because they differ in their metacognitive sensitivity (Galvin et al. 2003). To overcome this particular issue, Maniscalco and Lau (2012, 2014) proposed the “meta‐*d*′” (or relative type‐2 sensitivity), which takes into account the sensitivity in the type‐1 (objective) task to compute metacognitive sensitivity, thus assessing how well confidence ratings track task accuracy (i.e., the extent to which confidence ratings discriminate between the observer's own correct and incorrect stimulus classifications). More precisely, meta‐*d*′ computes the type‐1 (objective) performance that would be expected given the type‐2 (subjective) information if the participant was an ideal observer^10^ (Sherman, Barrett, and Kanai 2015). Its interpretation is quite straightforward: if meta‐*d*′ equals *d*′ (meta‐*d*′ = *d*′), then there is optimal metacognitive performance meaning that all information available for the type‐1 (objective) decision is also metacognitively (consciously) available. If instead meta‐*d*′ is lower than *d*′ (meta‐*d*′ < *d*′), one could assume that at least part of the information available for the type‐1 (objective) task is not available for the type‐2 (subjective) task, making the observer metacognitively inefficient, and part of the performance could be attributed to unconscious processes (see Maniscalco and Lau 2014 for a complete description of the process of calculating the meta‐*d*′). Despite its advantages, meta‐*d*′ has also some drawbacks. For example, it appears to be noisier (i.e., higher variance, especially when the number of trials per subject is low) than other SDT‐based measures and is not robust to large deviations from the SDT assumptions (Barrett, Dienes, and Seth 2013). In summary, these recently developed measures, although promising for capturing metacognition independently of response bias, should be employed and computed with caution, especially when few trials are collected (Sherman, Barrett, and Kanai 2015).

## 5 The Future Is Here: New Approaches and Challenges in Measuring Consciousness

In recent years, new approaches have been developed to solve some of the problems associated with measuring consciousness, while new and old challenges still persist. These new procedures offer promising research tools that might help consciousness researchers capture the elusive phenomenon of subjective experience. We discuss the most relevant of them below.

### 5.1 Evidence for the Null: Bayesian Approaches to Support the Absence of Awareness

A central part of consciousness research relies on determining whether a mental state is conscious or not. Inferring unconscious mental states using null hypothesis significance testing (NHST), however, implies affirming the null hypothesis (e.g., that some measure of conscious processing is at chance level), and it is already well‐known that absence of evidence does not imply evidence of absence. Indeed, within the frequentist statistical approach, a non‐significant result does not indicate evidence favoring the null hypothesis (e.g., absence of awareness), but only the absence of evidence favoring the alternative hypothesis (Dienes 2015). An useful alternative is Bayesian inference, which allows to quantification of the support for the null in comparison with the alternative hypothesis (Shanks, Malejka, and Vadillo 2021). Within consciousness research, this approach can be employed both with objective and subjective measures of awareness to evaluate the strength of evidence in support of the null, unconscious mental state.^11^

Interestingly, Bayesian modeling has recently been adapted to those approaches that infer unconscious processing using regression‐based strategies (Greenwald, Klinger, and Schuh 1995, see footnote 5; Goldstein, Sklar, and Siegelman 2022). In this method, the effect obtained in the indirect task is predicted in a regression model from centered awareness scores (where zero indicates the absence of awareness). The Bayesian generative modeling approach developed by Goldstein and colleagues allows to circumvent the main problem of the original method, which is the bias in the intercept estimation due to measurement error that leads to an overestimation of the unconscious effect (Goldstein, Sklar, and Siegelman 2022; Shanks, Malejka, and Vadillo 2021; see Jimenez et al. 2023, for an application of this approach to a masked priming paradigm). Bayesian approaches are becoming more popular and more widely adopted. Tsikandilakis et al. (2023), for example, recently proposed an alternative Bayesian method that determines unbiased individual unaware thresholds in the study of unconscious perception. This method is useful as it utilizes Bayesian analysis to explore the durations that provide evidence for chance‐level perceptual performance. Overall, the implementation of new approaches and Bayesian inference will play a crucial role in the future of consciousness research (see Dienes 2015, for a thorough review of the Bayesian approach to the study of (un)conscious processes; and Fisk and Haase 2023, for a recent comparison of frequentist and Bayesian approaches for studying unconscious perception).

### 5.2 Sensitivity Versus Awareness: General Recognition Theory and Model‐Based Analyses

In a recent study, Pournaghdali et al. (2023) proposed an innovative model‐based analysis to evaluate the relationship between consciousness and perceptual processing, expanding SDT to a multidimensional model called general recognition theory (GRT). The experimental procedure is very similar to what has been reviewed so far: participants perform two different tasks, a perceptual discrimination task and a subjective awareness task with at least two levels. Once the data is collected, the analysis estimates a GRT model for the data and constructs a sensitivity versus awareness (SvA) function that represents the sensitivity in the perceptual discrimination task as a function of the relative likelihood of awareness. These model‐based methods constitute a way to avoid some of the traditional flaws in consciousness research such as the post hoc data selection, the criterion bias, or the errors in the calculation of regression slope and/or intercept (Shanks, Malejka, and Vadillo 2021). More interestingly, they allow to go beyond the simple dissociation between conscious and nonconscious perception, and formally answer more general questions about the dependency of different types of processing on awareness, and to what extent they can occur in its absence (Pournaghdali et al. 2023). A similar approach was previously taken by Azzopardi and Cowey (1997) while evaluating unconscious perception in the blindsight patient G.Y. They employed SDT to mathematically model the expected relationship between a 2AFC task and a Yes/No awareness task, to get a non‐biased estimate of the sensitivity of the awareness task, controlling for criterion biases. The modeling of the relationship between two different tasks can be broadly extended to the field of consciousness research. According to this approach, once the relation between a given task, a particular awareness measure, and the factors affecting this relationship (e.g., attentional resources devoted to the task, characteristics of the awareness judgment, etc.) is understood, it might be possible to create models of this relationship given certain parameters. Next, we could use this modeling as a benchmark against which to compare the performance of the participants under different circumstances and experimental conditions, and subjected to different task and attentional demands. A good example of this approach is given by Michel (2023b) in relation to the use of metacognitive (type‐2) awareness measures: for a given task, we could map the relationship between type‐1 (perceptual) and type‐2 (metacognitive) performance under full visibility conditions. Once we know the relationship between these two tasks, we can estimate the expected type‐2 performance of an ideal observer and compare the expected performance with the actual performance under different visibility conditions and task demands.

### 5.3 Measuring the *Qualia*, the Qualitative Contents of Awareness

Much has happened since David J. Chalmers' accurate assertion “the development of more sophisticated methodologies for investigating first‐person data is the greatest challenge now facing a science of consciousness” (Chalmers 1999). Unfortunately, little progress has been made in developing methodologies for examining the qualitative contents of consciousness (i.e., *qualia*, as they are often called by philosophers) from the third‐person perspective of the scientific method. Traditionally, this type of question has been debated on philosophical grounds (e.g., the Phenomenology of Edmund Husserl; see Kockelmans 1994, for a review) but, in contrast, the scientific approach to the nature of the relationship between *qualia*, contents of consciousness, and behavioral reports has not yet been established as a consolidated area of research in the science of consciousness. As we have seen throughout this review, psychologists' and neuroscientists' strategies have focused on reducing reports of conscious experience to narrow response categories, either the typical two forced‐choice response or to specific labels in subjective awareness scales. However, *there is more to experience than meets the button* (Haun et al. 2017), and it is evident that the richness of visual experience has been neglected by researchers so far. Among these lines is the relevant work by Mattler, Albrecht, and colleagues (Albrecht and Mattler 2012; Koster, Mattler, and Albrecht 2020), who developed a novel method for examining the phenomenological experience of simple visual stimuli under visual masking. Their approach relies on spontaneous verbal reports of the presented stimuli (e.g., “at first, the target was small in the middle of the star of the mask and then grew in size to the border of the mask”) across various experimental conditions, enabling a direct mapping of the perceptual space of visual experience, even in situations with significantly reduced visual stimulation. Their findings support the idea that phenomenal consciousness is a multidimensional pattern not reducible to a single measure (Koster, Mattler, and Albrecht 2020). This multidimensionality could have significant implications; for example, any measure of consciousness that aims to be exhaustive (see Section 4.2) must capture the complex, multivariate pattern of visual experiences, which cannot be achieved using single one‐dimensional subjective or objective scales. Overall, it seems clear that our conscious experience is not *binary* nor *quaternary*; by contrast, it is derived by blending different inputs from several sensory modalities into a rich unified subjective experience (Tsuchiya, Saigo, and Phillips 2023). Undoubtedly, this is a complex issue, perhaps because it sits close to the “hard problem” of consciousness.

There have been other timid proposals among researchers to address the complex issue of investigating the *qualia*. Some promising contributions come from the research group led by Nao Tsuchiya. For example, introducing a massive report paradigm (MRP; Qianchen, Gallagher, and Tsuchiya 2022) the authors have aimed to assess the informativeness of consciousness by measuring how accurately an individual can distinguish between “what they saw” and “what they did not see.” Therefore, the MRP is an innovative approach to studying consciousness by capturing detailed reports of subjective experiences during a task (Qianchen, Gallagher, and Tsuchiya 2022). The use of natural stimuli is central within the MRP and allows researchers to collect rich data on the contents of consciousness. The approach is particularly useful for exploring the complexity and multidimensionality of conscious experience (e.g., the richness of conscious experience; Haun et al. 2017), as it goes beyond traditional, simplistic measures to capture a more nuanced picture of what individuals perceive during a task. By expanding both the number of reports and stimuli, the MRP aims to take an initial step toward estimating the true extent of informativeness in phenomenal consciousness.

Regarding the study of the intrinsic nature or *identity* of subjective experience, Tsuchiya, Taguchi, and Saigo (2016) have proposed, in the context of the Integrated Information Theory (IIT; Tononi 2004), a mathematical approach for understanding the structure of *qualia* based on the formalism called *category theory* (Mac Lane 1998). Category theory (CT) is a general theory of mathematical structures and their relations, and it could be a useful tool for assessing whether two distinct subjective experiences or *qualia* are similar and, crucially, in *what qualitative way* they are similar (Tsuchiya, Taguchi, and Saigo 2016). According to these authors, CT would have various advantages over other mathematical frameworks, as it has the potential to bridge informational structures in completely different domains, such as the domain of *qualia*, information, and neural activity (Tsuchiya, Phillips, and Saigo 2022). Ultimately, the aim of this novel approach is the introduction of powerful mathematical tools for the understanding of different cognitive processes, and in particular the structure of conscious experience and its connection to the informational structures within the brain. Tsuchiya, Taguchi, and Saigo (2016); Tsuchiya, Phillips, and Saigo (2022) and Tsuchiya and Saigo (2021) have proposed empirical research programs for studying similarities between the categories of *qualia*, but it is still too early to judge whether it is possible to derive predictions from this proposal (or even from IIT^12^) that can be tested in psychology or neuroscience laboratories.

### 5.4 The Case for Unconscious *Qualia*: The Brain‐Based Argument

A widely accepted view in the philosophy of mind, and cognitive science in general, considers the qualitative aspects of experience, the *qualia*, to be inherently conscious (Marvan 2024). Discussions of unconscious sensory qualities are only accepted when these unconscious *qualia* are attached with a different meaning, thus not being equal to the conscious sensory qualities. Among these lines, Rosenthal (2005) proposed a theory suggesting that unconscious mental qualities differ from those we consciously experience. Rosenthal's theory thus aligns with the common belief that unconscious qualities must be fundamentally distinct from conscious ones. While unconscious qualities might influence behavior and allow for perceptual discrimination, conscious qualities are defined by their subjective experience and the “what it's like” aspect. According to Rosenthal (2005), this conscious subjective experience would be produced by appropriate higher‐order thoughts.

The brain‐based argument (BBA) for unconscious sensory qualities, as proposed by Marvan (2024), shares two key ideas with Rosenthal's proposal. First, both suggest that mental qualities can exist in both conscious and unconscious states. Second, they agree that unconscious qualities become conscious through a separate process or mechanism. However, Marvan's BBA differs from Rosenthal's approach by asserting that unconscious sensory qualities can be *exactly the same* as conscious ones. According to Marvan, the same mental state, containing identical sensory qualities, can be either conscious or unconscious, depending on the conditions of perception.^13^ Therefore, while unconscious sensory qualities lack subjective awareness, they do not differ in their qualitative nature from conscious ones: Sensory qualities are entirely formed at an unconscious level, but they require interaction with a separate, nonqualitative process or mechanism to become consciously accessible to the subject. In the initial stage of perception, the mental state, including all its qualitative aspects, is established unconsciously. In the subsequent stage, these fully developed qualitative states are made consciously accessible to the subject through a distinct nonqualitative process or set of processes (Marvan 2024).

Following this view, consciousness research could strive to find different brain correlates associated with qualitative processes and consciousness‐conferring mechanisms in the brain. The first endeavor would focus on the processes that form sensory qualities, whether conscious or not, while the second would explore the nonqualitative mechanisms that confer consciousness (Marvan 2024). This division would impact the research into the neural correlates of consciousness, where the current methodology aggregates all correlated neural activity as “the NCC.” If the distinction between qualitative and consciousness‐conferring mechanisms is valid, it would help to identify the functions of different neural processes that contribute to experienced mental states. Another methodological insight from the BBA is that the part of the NCC responsible for the qualitative character of conscious mental states also plays the role of a neural correlate of *un*conscious sensory contents, elicited by the same stimuli as the conscious percepts. The distinction between qualitative processes and those that confer consciousness might make the challenge of explaining conscious qualitative states more manageable. Future research based on these insights from the BBA might prove to be useful in the search for the NCC.

### 5.5 Cracking the Neural Code: Can Neuroimaging Data Inform About Our Subjective Experience?

The recent introduction of machine learning‐based models for the analysis of neuroimaging data has opened new roads for the exploration of the anatomy of consciousness (Haynes 2009). This approach promises to assess how conscious experience and its contents are encoded in the brain from distributed patterns of brain activity. *Encoding* means that there is a stable mapping between the states of NCC and conscious experiences (Haynes 2009). Thus, *encoding* models aim to predict brain response patterns from descriptions of the experimental conditions (Naselaris et al. 2011). In other words, the *encoding* approach attempts to characterize how brain activity varies when there is concurrent variation in the sensory stimulation, the cognitive state, or the action. Complementarily, *decoding* models make an inverse encoding, whose objective is not to model brain information processing, but to “crack the neural code” for testing whether a brain region represents a particular kind of information. In other words, decoding analyses unravel the products or contents but not the process of brain computation (see Kriegeskorte and Douglas 2019, for a review). Although the use of the decoding models is still very recent in consciousness science, it has already yielded interesting results that provide confidence in its future research potential. For example, the fMRI decoding by Hatamimajoumerd et al. (2022) have observed global activation across the brain when participants report their conscious experiences but, without reports, the frontal lobe is not activated. However, visibility of the stimuli (i.e., differential activation between conscious and unconscious trials) could still be decoded in the frontal lobes even in the absence of an overt report. Interestingly, recent advances from studies exploring semantic decoding from brain recordings (e.g., Tang et al. 2023) might pave the way for the future study of *qualia*. Tang et al. (2023) introduced a novel non‐invasive decoder that reconstructs continuous language from cortical semantic representations recorded using fMRI. Given novel brain recordings, this decoder generates intelligible word sequences that recover the meaning of perceived speech, imagined speech, and even silent videos. This can be indeed characterized as a brain–computer interface capable of decoding continuous language from non‐invasive recordings. Following these findings, one could think that we are closer to the introduction of a novel brain‐computer interface that decodes conscious states (a “consciousness‐meter”) or even subjective experiences (a “*qualia*‐meter”) from noninvasive recordings. Time will tell whether these types of “*mental*‐*meters”* remain confined to the pages of science fiction books, or eventually make their way to the pages of scientific publications.

Machine‐learning‐based decoding models will not only produce relevant findings in the study of conscious experience but they are already being implemented in the study of unconscious processing. A recent proposal suggests that brain‐based measures may be used to pinpoint the presence of unconscious knowledge associated with null perceptual sensitivity (Soto, Sheikh, and Rosenthal 2019). This approach aims to isolate the representation of unconsciously processed items by conducting fine‐grained analyses of brain activity patterns, especially when behavioral measures show null sensitivity. Some methods to reveal the brain's representation of unconsciously processed information include using transfer learning, where models pretrained on conscious items are repurposed to characterize unconscious knowledge, as well as analyzing the differences between brain representations of conscious and unconscious items using computational models (Soto, Sheikh, and Rosenthal 2019). Within these lines, relevant results have been obtained by decoding of BOLD responses by Mei, Santana, and Soto and colleagues (2022), which showed that unconscious contents (i.e., categories of living and nonliving images) can be decoded from multi‐voxel patterns that are highly distributed alongside the ventral visual pathway and also involving parieto‐frontal regions. This finding is apparently contradictory to the Global Neuronal Workspace Theory of consciousness (Dehaene 2014; Dehaene and Naccache 2001), which predicts that unconscious contents only activate primary sensory cortices and that global broadcasting of information through the brain only is possible for conscious contents.

A complementary line of research explores the potential dissociations between subjective and objective measures of visual awareness based on their neural underpinnings (di Gregorio et al. 2022; di Luzio et al. 2022; Trajkovic et al. 2023; Mazor, Dijkstra, and Fleming 2022). By applying decoding analyses to functional brain imaging data, Mazor, Dijkstra, and Fleming and colleagues (2022) have recently shown that prefrontal representations of subjective visibility are contaminated by neural correlates of decision confidence. These findings highlight the importance of controlling for confidence when investigating reports of awareness versus unawareness, for which new analysis methods, which factor in the metacognitive aspects of awareness need to be implemented. Innovative approaches using electrophysiological recordings (EEG) and rhythmic transcranial magnetic stimulation (rhythmic‐TMS) have recently provided evidence of a double dissociation between the alpha frequency and alpha amplitude of the EEG, linking the former to spatiotemporal sampling resources and the latter to the internal, subjective representation and interpretation of sensory events (di Gregorio et al. 2022).

These recent works are representative examples of the promising future that innovative brain‐based approaches may have within consciousness research in the coming decades.

## 6 Conclusions

The study of consciousness is considered by many one of the most difficult contemporary scientific endeavors and confronts several methodological and theoretical challenges. In the present article, we reviewed the many challenges faced by consciousness research since awareness and subjective experience entered the lab in the late 19th century. Aside from comprehensively exploring the two main approaches within the field—objective (i.e., performance based) and subjective (i.e., report based)—measures of awareness, we have additionally discussed several new approaches (such as Bayesian inference to support the absence of awareness or new machine‐learning based decoding models) as well as future challenges (e.g., measuring the *qualia*) that the science of consciousness faces in the near future.

Undoubtedly, the problem of measuring consciousness is central for the advancement of consciousness research, as there is a pressing need for reliable and valid measures of awareness to test the hypotheses and predictions derived from neuropsychological theories that attempt to explain consciousness (see Seth and Bayne 2022, for a recent review of the main theories of consciousness). Galileo Galilei is often attributed with the quote “measure what can be measured and make measurable what cannot be.” This goal has guided scientific exploration for centuries and it has not always been easy to make reality measurable. Our current challenge is to make measurable our own subjective reality, which is paradoxically, the *only reality we experience*. The path ahead is both challenging and exciting in the relatively young science of consciousness.

## Author Contributions

**Mikel Jimenez:** conceptualization (equal), supervision (equal), writing – original draft (equal), writing – review and editing (equal). **Antonio Prieto:** conceptualization (equal), supervision (equal), writing – original draft (equal), writing – review and editing (equal). **José Antonio Hinojosa:** conceptualization (equal), supervision (equal), writing – review and editing (equal). **Pedro R. Montoro:** conceptualization (equal), supervision (equal), writing – original draft (equal), writing – review and editing (equal).

## Conflicts of Interest

The authors declare no conflicts of interest.

## Related Wires Article

Confidence in consciousness research

## Data Availability

Data sharing is not applicable to this article as no new data were created or analyzed in this study.

## Endnotes

^1^
Since the visual system is the most studied and better understood of our sensory systems, and well tested experimental techniques (e.g., visual masking, continuous flash suppression) can be used to “switch” a stimulus visible or invisible under controlled lab conditions (see Breitmeyer 2015; Kim and Blake 2005, for a review), most of the work on the search for the NCC has studied visual awareness.

^2^
The term “subliminal” was first introduced by Johann Herbart (1776–1841) to describe ideas that competed beneath the threshold of consciousness. Hermann von Helmholtz (1821–1894) proposed that visual perception predominantly resulted from non‐conscious inferential processes, and Gustav Fechner (1801–1887) introduced psychophysical methodologies that established a rigorous connection between the physical attributes of stimuli and psychological experiences.

^3^
Note that different authors (Cheesman and Merikle 1984, 1986) still endorsed the use of subjective measures of awareness. Given that consciousness is a subjective state—these authors argued—subjective thresholds should be a better criterion than objective thresholds to distinguish between conscious and non‐conscious processing.

^4^
Post hoc data selection, which involves analyzing data only from participants whose awareness scores fall below a specific cut‐off (e.g., chance level in the prime visibility task). The mean performance score is then calculated for this subset of “unaware participants.” If significant priming effects are observed in this subgroup, it is concluded that true unconscious perception has occurred. However, the use of this solution is controversial and strongly discouraged (Rothkirch et al., 2022; Shanks 2017), because it introduced a statistical phenomenon known as *regression to the mean* (RttM). Essentially, selecting a subsample of participants based on their awareness and using the selected awareness values to compute the mean awareness in this subgroup leads to “double‐dipping” (i.e., using the same measure twice), which introduces a bias because the measurement error is no longer randomly sampled within the subgroup (Rothkirch et al., 2022). Debate on the RttM is ongoing. Yaron et al. (2023) recently proposed a non‐parametric solution to the RttM problem, while new Bayesian approaches (Goldstein, Sklar, and Siegelman et al., 2022) also aim to address this challenge (see section 5.1. for a discussion).

^5^
Jacoby's process dissociation procedure (PDP) (1991) represents a significant landmark in investigating the disparities between implicit and explicit processing. Jacoby argues that both direct and indirect measures can be subject to contamination from unconscious and conscious influences, respectively. The PDP seeks to disentangle and quantify the relative contributions of these conscious and unconscious influences on performance. This approach proposes an alternative to the traditional dissociation logic by exploring the relative sensitivity of measures and differentiating the effects of conscious and unconscious influences on performance. Additionally, they acknowledge the potential for contamination in both direct and indirect measures, shedding light on the complex interplay between implicit and explicit processing. Greenwald, Klinger, and Schuh (1995) proposed a regression‐based method to avoid any post hoc data selection. In this approach, the unconscious priming effect is predicted using a linear regression model, where the prime awareness values are centered by subtracting the chance level from the actual performance values. This centering ensures that a value of zero indicates no awareness of the prime. The regression intercept represents the predicted priming effect for a hypothetical participant with awareness performance at chance level in a forced‐choice measure. However, several studies have demonstrated that the Greenwald regression tends to produce biased estimates of the unconscious effect, overestimating the intercept value (Dosher, 1998; Klauer et al., 1998; Sand & Nilsson, 2016). Recently, new methods have been proposed to overcome the biases in the Greenwald regression (Goldstein, Sklar, and Siegelman 2022) which are discussed in Section 5.1.

^6^
The PAS has been compared to other different length subjective scales. Results have shown that those scales of greater length and unlabeled intermediate ratings are less sensitive to intermediate awareness experiences and tend to produce more dichotomously distributed subjective ratings (Pretorius, Tredoux, and Malcolm‐Smith 2016). In addition, the PAS has been compared to two other types of scales, confidence ratings and post‐decision wagering (Sandberg et al. 2010; see further discussion in Section 4.3). The purpose of using these other types of scales is often to avoid participants relying on overt introspection of their experience, as participants may not be very good at that. However, the experiment found PAS to be superior to the other scales in three ways. First, the accuracy is closer to chance when participants used the lowest rating on PAS compared to when participants used the lowest scale step on the confidence and wagering scales, thus indicating that the competing scales overestimated the amount of unconscious processing. Second, the correlation between awareness and task accuracy was higher for PAS than for the other scales. This correlation is typically taken as an indicator of awareness. Third, the correlation between awareness and accuracy was more stable across different stimulus intensity conditions for PAS than for the other scales, which may be taken as evidence that the scale was used in a more consistent manner.

^7^
Different strategies have been proposed to address this particular issue. Clifford, Arabzadeh, and Harris (2008) proposed adapting the pay‐off matrix individually for each subject. Schurger and Sher (2008) on the other hand, suggested an alternative means to counter loss aversion by fixing the amount wagered and therefore the potential losses. Finally, Dienes and Seth (2010a) introduced a “no‐loss gambling” method thought to be insensitive to risk aversion.

^8^
According to Kiefer and Kammer, the relation between thresholds derived from subjective and objective measures of awareness may fit one of three possible scenarios. Under the first one, subjective thresholds lag behind objective thresholds. An earlier threshold for objective performance could be partially explained on fast unconscious processing (which would produce above‐chance discrimination performances), while subjective measures would require longer lasting visual consolidation giving rise to a specific phenomenal experience. Alternatively, it might be the case that only subjective, but not objective measures depend on meta‐cognitive evaluations, which includes an extra processing step. Under the second scenario, objective measures would lag behind subjective measures, because subjective measures capture a broader range of sensory experiences compared to objective measures. Finally, under the third scenario, subjective and objective measures would converge (see Kiefer and Kammer 2024, for a discussion).

^9^
It is important to note that although researchers are usually concerned with how a confidence report reflects a first‐order percept or decision (which does not require a phenomenal experience itself), confidence reports could also have their specific phenomenology (*qualia*), regardless of what they are about (Koriat 2007; Morrison 2016). Therefore, some authors have proposed that “decisional confidence” could be a legitimate target for studying how qualitative experiences emerge (see Peters 2022, for a detailed discussion on metacognitive processes as a tool for studying subjective phenomenology).

^10^
This question is related to the classical distinction in the metacognitive literature between absolute and relative accuracy (Dunlosky and Metcalfe 2009; Fleming and Lau 2014). We can imagine two observers that have different type‐1 sensitivities (e.g., d’_1_ = 2, and d’_2_ = 1), but both make an optimal metacognitive use of the type‐1 information that is available for them (which is not the same for both, given their different type‐1 sensitivities) to perform the type‐2 task. In this situation, the confidence ratings of the first observer (d’_1_ = 2) will be, overall, more predictive of the accuracy in the type‐1 task (i.e., the observer has a greater “absolute” type‐2 sensitivity). However, this greater sensitivity is driven only by the differences in the available information for the type‐1 task between the two observers, not the quality of the metacognitive evaluation itself. If we want to evaluate their “relative” sensitivity (i.e., the efficacy of the metacognitive mechanisms themselves), we need to evaluate the observed type‐2 sensitivity in relation to the expected (maximum) sensitivity given the observed type‐1 performance. Although there are different approaches for the estimation of the relative type‐2 sensitivity (see Galvin et al. 2003), Maniscalco and Lau (2012) proposed to express it as a function of the type‐1 distribution or, in other words, as a measure of the (type‐1) signal available to perform the type‐2 task, which they call meta‐d’.

^11^
In the case of the metacognitive approach and subjective (type‐2) measures, one of the criteria for knowledge to be unconscious is that participants inform they are guessing (or having no visual experience) and, nonetheless, they classify above chance (Dienes 2007). This constitutes the so‐called *guessing criterion*, according to which unconscious processing is signaled by a significant result (the above chance performance in the type‐1 task). The risk, however, is that we reject the existence of unconscious processing if we found the type‐1 (objective) task does not exceed the performance expected by chance. A second criterion commonly employed when dealing with subjective measures is the *zero*‐*correlation criterion* (Dienes 2007). According to this, unconscious processing is revealed by the absence of a significant relation between confidence (or visual experience) and accuracy. Contrary to the *guessing criterion*, the problem here is to take unconscious processing for granted just because our data is insensitive to the relationship between the type‐1 and type‐2 tasks (Dienes 2015), a situation where a Bayesian approximation to data analysis is again necessary to quantify the support for the null hypothesis and the existence of unconscious processing.

^12^
See Lau (2023), for a recent controversy regarding the scientific nature of the Information Integrated Theory of consciousness.

^13^
According to Marvan (2024), the brain‐based argument (BBA) for unconscious sensory qualities could more accurately be referred to as the argument from the shared neural resources of conscious and unconscious sensory qualities. The BBA is based on the following points: (a) Specific brain areas are functionally specialized for processing certain types of visual content; (b) These specialized brain areas are directly involved in generating this type of content in conscious experience; (c) The same brain areas are activated in a similar way when the same visual stimulus is present, even if the subject is not consciously aware of it; and (d) Conscious visual content has a qualitative nature. Based on these premises, the BBA concludes that unconscious visual content, being produced in the same manner and in the same brain areas as conscious content, shares the same qualitative nature as the conscious visual content. Main objections to this account refer to points b (e.g., the specialized brain areas are not sufficient for generating a conscious experience) and c (e.g., same brain areas are only weakly activated when the stimulus is present but not consciously perceived; see Marvan 2024, for a thorough discussion on the criticism to the BBA).
